# Structural and Functional Regulation of RyR2 in Cardiac Calcium Handling and Arrhythmogenesis

**DOI:** 10.3390/biomedicines14030662

**Published:** 2026-03-14

**Authors:** Kaiyang Gao, Wenzhuo Wang, Yanan Ling, Baihe Li, Chenlei Xing, Nike Li, Xiaolan Yin, Lan Tao, Xiaoqing Li, Junling Qiu, Xuanqi Wang, Jinhong Wei

**Affiliations:** 1 Department of Cardiology, Northwest University First Hospital, Xi’an 710043, China; 202324241@stumail.nwu.edu.cn (K.G.); 20255062@nwu.edu.cn (J.Q.); 2 School of Medicine, Northwest University, 229 Taibai North Road, Xi’an 710069, China; 202422200@stumail.nwu.edu.cn (W.W.); 202522350@stumail.nwu.edu.cn (Y.L.); 202324232@stumail.nwu.edu.cn (B.L.); 202434414@stumail.nwu.edu.cn (C.X.); 2022113135@stumail.nwu.edu.cn (N.L.); 202522348@stumail.nwu.edu.cn (X.Y.); 202332651@stumail.nwu.edu.cn (L.T.); xiaoqingli@stumail.nwu.edu.cn (X.L.)

**Keywords:** ryanodine receptor 2, cardiac arrhythmias, heart rate regulation, excitation–contraction coupling, calcium homeostasis

## Abstract

Cardiac Ca^2+^ handling is critical for excitation–contraction coupling (ECC), with the ryanodine receptor type 2 (RyR2) serving as the key sarcoplasmic reticulum (SR) Ca^2+^ release channel in cardiomyocytes. The dysfunction of RyR2 is linked to fatal cardiac arrhythmias, including heart failure (HF) and catecholaminergic polymorphic ventricular tachycardia (CPVT). This review aims to elucidate the structural basis of RyR2, its core role in cardiac ECC and Ca^2+^ homeostasis, and the regulatory mechanisms of key modulators on its activity. By integrating recent high-resolution cryo-EM structural analyses with molecular and cellular studies on RyR2 regulation, as well as clinical evidence of RyR2 mutations in arrhythmogenic heart diseases, we provide a comprehensive overview of the field. Cryo-EM has unraveled RyR2’s gating mechanisms, ligand-binding sites, and structural features. Functionally, RyR2 mediates calcium-induced calcium release (CICR) and maintains Ca^2+^ homeostasis through coordination with SERCA2a and NCX. Key modulators (CaM, FKBP12.6, and PKA/CaMKII) and disease-linked mutations regulate RyR2 activity through distinct pathways, with defective RyR2 leading to store-overload-induced Ca^2+^ release (SOICR) and arrhythmias. Furthermore, reactive oxygen species (ROS) can induce RyR2 oxidation, establishing a pathological Ca^2+^ leak-ROS cycle in heart disease. In conclusion, RyR2 is a pivotal sensor of myocardial function, with its structural and regulatory mechanisms now well-characterized by recent studies. However, the effects of numerous RyR2 mutations remain unclear, and deeper mechanistic insights will lay a key foundation for developing novel therapies against RyR2-related cardiac diseases.

## 1. Introduction

As a ubiquitous second messenger, Ca^2+^ is critical in the coupling between excitation and contraction. A large body of research have now confirmed that Ca^2+^ signaling and handling in cardiac muscle is a cycling process that involves the cooperative changes in extracellular Ca^2+^, cytosolic Ca^2+^, and Ca^2+^ stored in organelles such as the sarcoplasmic reticulum (SR) and mitochondria. Abnormal Ca^2+^ handling in the heart has been implicated in a variety of pathological conditions including cardiac arrhythmias, cardiomyopathies, and heart failure (HF) [1,2,3]. The major processes and modulators involved in Ca^2+^ handling during this excitation–contraction coupling (ECC) process are discussed in the following sections.

Five types of voltage-gated Ca^2+^ channels have been identified, two of which (L- and T-type) are abundantly expressed in cardiomyocytes. The L-type Ca^2+^ channel (LTCC) is critical for the initiation of Ca^2+^-induced calcium release (CICR) because of its large single channel conductance, long opening time, and slow voltage and Ca^2+^-dependent inactivation [4]. After membrane depolarization during an action potential (AP), the LTCC is activated. The resultant Ca^2+^ influx increases the Ca^2+^ level within a restricted area between the sarcolemma and the SR where RyR2 is located. In this restricted area, which is termed a “junctional zone” or “dyadic cleft”, the free Ca^2+^ concentration can rise from a resting level of 100 nM to as high as 10 µM [5], facilitating the activation of RyR2.

The ryanodine receptor (RyR) and inositol trisphosphate receptor (IP3R) are the two predominant forms of Ca^2+^ release channels located in the SR. They share a certain structural homology and functional similarities [6]. However, the activation of IP3R requires not only Ca^2+^ but also inositol trisphosphate (IP3). Consequently, its role in myocardial calcium regulation is considered minor. In contrast, RyR is activated by a local increase in the Ca^2+^ level and results in a large Ca^2+^ release from the SR, contributing to the initiation of muscle contraction. The altered RyR function disturbs the Ca^2+^ homeostasis and could cause an aberrant ECC. The structure and function of RyR2 will be introduced in detail in the following sections.

The inactivation of the LTCC is mediated prominently by Ca^2+^ but also by membrane repolarization [7]. When the cytosolic Ca^2+^ increases, the free Ca^2+^ binds to calmodulin (CaM), which is an important modulator of multiple protein targets including the LTCC and RyR2. The Ca^2+^–CaM interacts with the C-terminal region of the LTCC and causes the inactivation of the channel [8,9]. Therefore, the Ca^2+^-dependent inactivation allows for effective autoregulation and terminates the Ca^2+^ influx via the LTCC.

To ensure a comprehensive review, we systematically searched the PubMed database for relevant studies. The search strategy combined keywords and MeSH terms, including “Ryanodine Receptor 2/(RyR2),” “cardiac calcium handling,” “excitation-contraction coupling/(ECC),” “calmodulin/(CaM),” “FKBP12.6,” “PKA,” “CaMKII,” “catecholaminergic polymorphic ventricular tachycardia/(CPVT),” and “heart failure/(HF).” The search focused on English-language articles published over the last 20 years, with particular attention to high-resolution cryo-EM structural studies and key clinical findings. The reference lists of the included articles were also manually screened to identify additional relevant publications.

## 2. Structural Framework of RyR2: From Cryo-EM Architecture to Functional Implications

Three isoforms of RyRs (RyR1, RyR2, and RyR3) have been identified in mammalian tissues [10]. Among them, RyR2 is the predominant isoform expressed in the heart, whereas RyR1 is abundant in skeletal muscle [11,12]; both play important roles in ECC. RyR3 is mainly found in organs such as the brain, kidney, and liver, and is involved in a variety of physiological processes [13,14]. The RyRs family controls the largest intracellular Ca^2+^ release channel located in the SR. Another intracellular Ca^2+^ release channel is the inositol 1,4,5-trisphosphate receptor (IP3R) [15,16]. However, in cardiomyocytes, IP3R plays virtually no role. The research of the structure of RyRs first started with RyR1 [17], RyR1 is an unusually large tetrameric ion channel in the SR and is a major component of the triadic junction at the site of ECC. Its three-dimensional structure was initially determined using cryo-electron microscopy, which identified three major classes of four-fold symmetric images and enabled three-dimensional reconstructions of two of them, though at a limited resolution [18].

**Figure 1:**
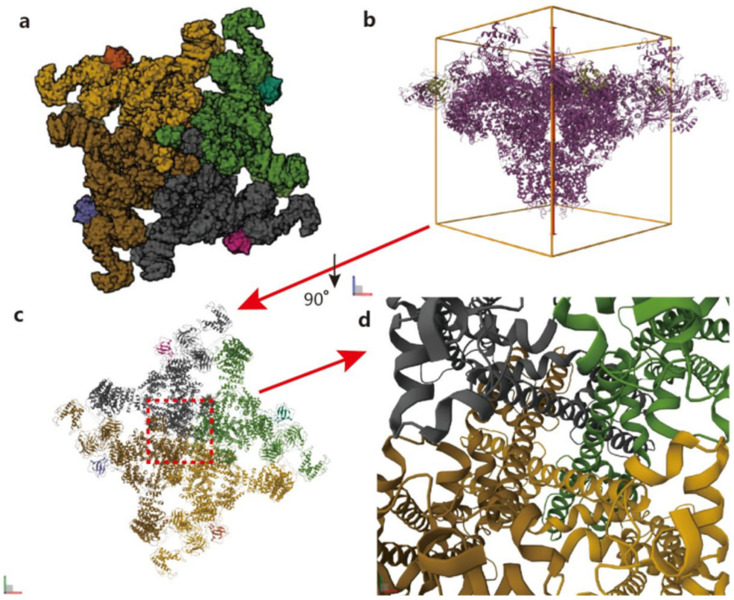
Structure information of RyR2 in the PDB database, PDB entry is 6JH6. (**a**) shows the RyR2 structure of *Homo sapiens* with a resolution of 4.80Å. The image obtained by cryo-electron microscopy was processed; (**b**) is the 3D structure of (**a**); (**c**) is a 90° downward rotation of (**b**); and (**d**) is a partially enlarged image of the red area in the middle of (**c**).

It was not until early 2015, with the advent of the cryo-electron microscopy revolution [19], that structural biology was transformed, achieving resolutions in the range of 3.8 Å to 6.1 Å [20]. These technological and software advancements enabled a deeper understanding of the connection between RyR2 structure and function. The resulting high-resolution images of RyRs channels have been instrumental in elucidating the mechanism of channel activation. Subsequently, the structural information of the open RyR1 channel and its closed state was obtained using the above technique [21,22,23]. In 2016, Peng et al. published a study in *Science* that further revealed the structural basis of the RyR2 gating mechanism and gave a structural comparison between open and closed RyR2 [24]. In 2019, Gong et al. reported in *Nature* the cryo-electron microscopy structure of RyR2 under eight different conditions, further revealing the receptor regulation mechanism of RyR2 [25]. Based on the above information, we now know the location, structure, and composition of the RyR2 channel in the cell and the mechanism involved [26], which will help us to better understand the structure of RyR2 and to further elucidate the mechanism of Ca^2+^ release during ECC in cardiomyocytes (see Figure 1).

RyRs, the major component of the triadic junction, are located in the interstitial region between the SR and the transverse tubule (T-tubule), and the RyRs receptor was purified from a pool of skeletal muscle SR junction terminal. The RyRs receptor was stabilized by solubilization with CHAPS and the addition of phospholipid. The purified product was examined by electron microscopy and found to be morphologically identical to the foot structure, which is called the junctional channel complex (JCC) because it contains a Ca^2+^ release channel, and consists mainly of an oligomer of a single high-molecular-weight protein [27,28]. RyRs are homotetrameric channels consisting of four subunits, each of which has ~5000 amino acids and fold into a cytoplasmic region and a transmembrane region [29,30].

RyR2 is located in cardiac myocytes and controls the release of Ca^2+^ from the largest intracellular calcium reservoir; thus, it is also known as the “SR calcium release channel” and belongs to the highly conserved family of the Ca^2+^ release channel [31,32]. Recent breakthroughs on the 3D structure of RyR2 have provided valuable insights into the structural–functional relevance of RyRs. In the recently resolved 3D structure, each RyR2 subunit contains a cytoplasmic region including the NH2-terminal region and the central domain. In the side views, these two parts and the channel domain are at the center of the channel and build up a structure called the “central tower”. In the central tower, the NH2-terminal region is on “top”, and the channel domain is at the “bottom,” with the connecting central domain in the middle [30,33]. Each RyR2 subunit has a CaM binding site, FK-506 binding protein (FKBP12.6), also known as calstabin2 (because it stabilizes the closed state of the RyR2 channel), PKA, sorcin, and phosphatases 1 and 2A (PP1/PP2A); RyR2 activity is regulated by Ca^2+^, Mg^2+^, and ATP; and covalent modifications can also affect RyR2 activity, such as phosphorylation, oxidation, and nitration. RyR2 function is also affected by a number of pharmacological agents, including caffeine, ruthenium red, the immunosuppressive drug FK506, and rapamycin [34].

This detailed structural understanding of RyR2, including the architecture of its cytoplasmic and transmembrane regions, the central tower configuration, and the binding sites for key modulators such as CaM, FKBP12.6, and PKA, provides a critical framework for interpreting the complex functional regulation of the channel. In the following sections, we will explore how these structural features enable RyR2 to participate in the ECC cycle (Section 3) and how various modulators and post-translational modifications dynamically regulate the channel activity (Section 4, Section 5 and Section 6).

## 3. The Excitation–Contraction Coupling Cycle

The heart is the center of blood circulation in the human body. As a complex pump, it drives blood circulation, supplies blood to body organs, and pumps blood through regular cyclic systolic and diastolic motions, in which Ca^2+^ plays an important regulatory role. Ca^2+^, as a ubiquitous second messenger, is essential for ventricular contraction and relaxation, the process of ECC.

Cardiac ECC is the process that links electrical excitation at the membrane surface to cardiac contraction (driving blood outflow) [35], in which Ca^2+^ is essential and myocardial contractility is regulated by cytosolic free Ca^2+^ [36]. First, the depolarization generated by the action potential activates LTCC located on the sarcolemma and in T-tubules. The resulting entry of a small amount of Ca^2+^ causes a large increase in cytosolic free Ca^2+^ in the dyadic space (the area surrounded by T-tubule and SR), and the increase in cytosolic free Ca^2+^ opens up the SR Ca^2+^-release channel RyRs, which causes the SR to release more Ca^2+^, a process known as CICR [37]. The combination of Ca^2+^ influx through LTCC and the Ca^2+^ release from SR raises the level of cytosolic free Ca^2+^, and enables Ca^2+^ to bind to troponin. This binding triggers myofilament sliding, leading to sarcomere shortening and muscle contraction.

**Figure 2:**
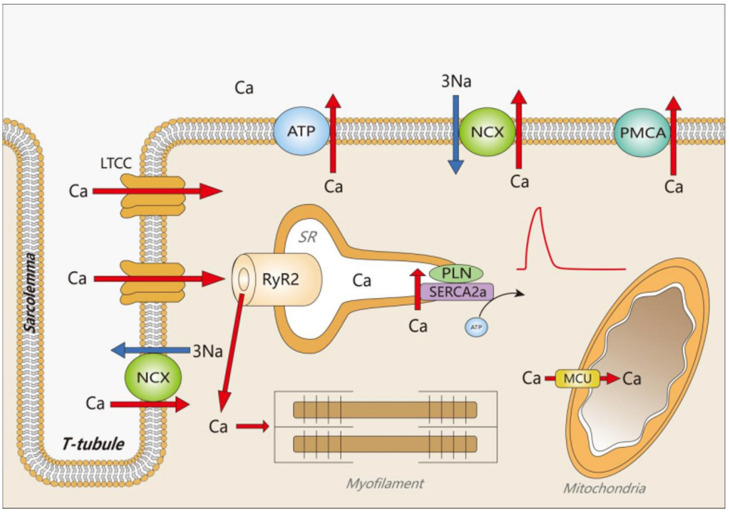
Excitation–contraction coupling mechanism and Ca^2+^ homeostasis. In cardiomyocytes, action potentials cause an excitatory contraction of the cardiomyocytes. The induced depolarization further opens the LTCC, resulting in massive Ca^2+^ entry into the cell and activation of RyR2 channels, leading to an increase in the concentration of free Ca^2+^ in the cytoplasm, which binds to troponin, triggering myofilament sliding, myonule shortening and muscle contraction. To enter diastole, free Ca^2+^ leaves the cytoplasm in several ways: SR Ca^2+^-ATPase, sarcolemmal Ca^2+^-ATPase, sodium–calcium exchange (NCX), or it is taken up by other organelles such as mitochondria.

After each contraction, cells need to enter diastole quickly, which requires the closure of RyRs in the SR [38], followed by the removal of Ca^2+^ from the cytoplasm [39,40,41,42]. The diastolic phase of ECC is not merely a passive relaxation process, but an energy-consuming process. During this period, two main processes occur within the cell. First, the cell returns to its resting potential state mainly under the action of the sodium-potassium pump (Na^+^/K^+^-ATPase, NKA). Second, cytosolic free Ca^2+^ gradually declines. The cytosolic Ca^2+^ level reduces as a large portion of Ca^2+^ is taken back into the SR by the SR Ca^2+^-ATPase (SERCA2a). The remainder of the Ca^2+^ is mainly extruded to the extracellular space through the Na^+^/Ca^2+^ exchanger (NCX) and the plasma membrane Ca^2+^-ATPase (PMCA). A small portion is taken into the mitochondria via the Ca^2+^ uniporter (see Figure 2) [3,43]. During muscle relaxation, 92% of cytosolic Ca^2+^ returns to the SR store in rat and mouse ventricular myocytes, while 7% is extruded by NCX and 1% via PMCA and mitochondria. In humans and some other mammals such as rabbits and dogs, SERCA contributes to 70% of Ca^2+^ removal, with 28% by NCX and 2% via PMCA and mitochondria [3]. The end result is a reduction in cytosolic free Ca^2+^ from 10 μM to 0.1 μM, allowing the myocardium to actually diastole and prepare for the next contraction [42,43].

Under physiological conditions, normal cardiac function requires cytoplasmic free Ca^2+^ to be sufficiently high during systole and low during diastole. RyRs play a key role in Ca^2+^ regulation in the ECC [44]. As mentioned above, the activation of the RyR2 channel greatly increases cytoplasmic free Ca^2+^ during systole; therefore, the channel should be turned off quickly when the cell enters diastole. Inappropriate Ca^2+^ handling by cardiomyocytes leads to myocardial contractile dysfunction, which triggers the development of various cardiomyopathies, including heart failure (HF), atrial fibrillation, ventricular fibrillation, catecholaminergic polymorphic ventricular tachycardia (CPVT), congenital long QT syndrome, and hypertrophic cardiomyopathy [45,46,47,48,49]. It has already been shown that these disorders are inextricably linked to RyR dysfunction. This review focuses on the structural information of RyR2 and its functional modulation.

## 4. Molecular Regulation of Ca^2+^ Homeostasis

Ca^2+^ is pumped from the cytoplasm into the SR by the action of SERCA2a located on the SR membrane [50], which is an energy-consuming active transport. This process is regulated by phosphoprotein (PLB), which inhibits SERCA2a when it is unphosphorylated and loses its inhibitory effect when phosphorylated [51,52]. The overexpression of SERCA2a was found in a rabbit model of atrial fibrillation to increase Ca^2+^ uptake by the SR, thereby decreasing cytosolic free Ca^2+^ [53]. In addition to this, histidine-rich Ca^2+^ binding protein (HRC), a Ca^2+^-buffering protein present in the SR, can also interact with SERCA2a. It has been found that the myocardial SR Ca^2+^ uptake rate was decreased in HRC-expressing mice, suggesting that HRC might have an inhibitory effect on SERCA2a [54].

NCX plays an essential role in the regulation of Ca^2+^ homeostasis and cardiac muscle contractility by mediating the electrogenic counter transportation of three Na^+^ for one Ca^2+^ across the plasma membrane. Under physiological conditions, the primary function of NCX in the heart is to extrude Ca^2+^, reduce the cytosolic Ca^2+^ level, and contribute to muscle relaxation, and the direction of exchange depends on the electrochemical gradient of the two ions [55]. During the resting potential of cardiomyocytes, the extracellular Na^+^ concentration is high, and NCX has a low affinity for the low concentration of intracellular Ca^2+^. Therefore, at this time, NCX typically imports three Na^+^ into the cell and extrudes one Ca^2+^. However, with the opening of the voltage-gated Na^+^ channel (Nav1.5), the cardiomyocyte rapidly enters into an AP from the resting potential.

**Figure 3:**
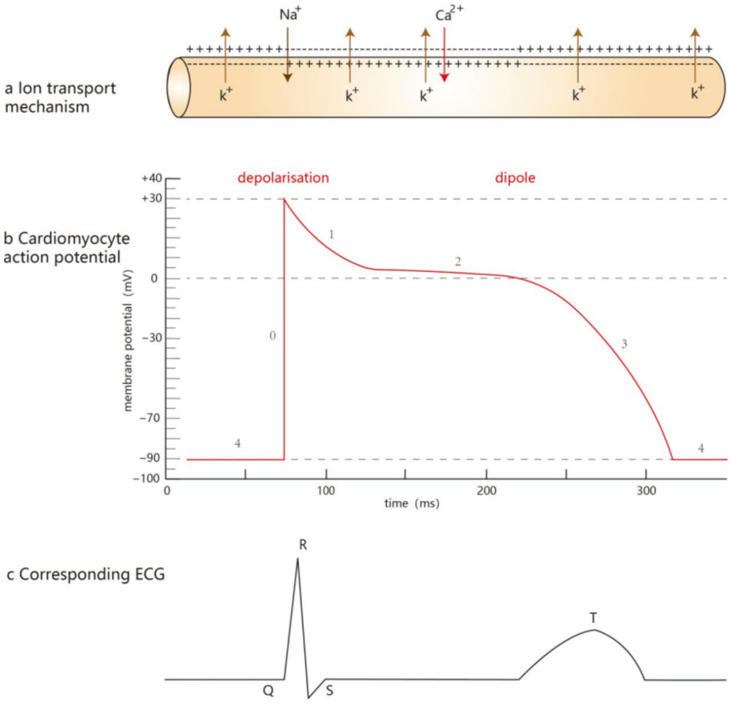
Ionic basis of cardiomyocyte action potential and its corresponding ECG waveforms. (**a**) Ion transport mechanisms underlying the cardiomyocyte action potential; (**b**) phasic characteristics of the cardiomyocyte action potential; and (**c**) corresponding surface ECG waveform. Phase 0 is caused by the rapid influx of Na^+^ due to the opening of the fast Na^+^ channel, resulting in a very rapid rise and extremely sharp waveforms. Phase 1 is the rapid repolarization phase, mediated by K^+^ efflux. Phase 2 is the plateau phase, in which Ca^2+^ influx and K^+^ efflux form a transient equilibrium, and the plateau phase is specific to cardiomyocytes, distinctly different from that of skeletal myocytes and neurons. The plateau phase prevents tonic contraction of the myocardium, which is important for maintaining normal cardiomyocyte function. Phase 3 is the late repolarization phase: Ca^2+^ inward flow stops, K^+^ outward flow continues, and the membrane potential eventually returns to −90 mv. Phase 4 maintains the membrane potential at the resting level under the combined action of Ca^2+^, K^+^, and Na^+^.

During phase 0 of the AP, Na^+^ flows into the cell through Nav1.5, causing the cell to undergo rapid depolarization. As a result, the local Na^+^ concentration near the plasma membrane rises transiently. The “forward mode” of NCX, which is “three Na^+^ in, one Ca^2+^ out” is inhibited; the “reverse mode”, which is the discharge of Na^+^ from the cell and the uptake of Ca^2+^, is activated, which means that Ca^2+^ is taken up while Na^+^ is discharged from the cell. However, this situation does not last for a long time, as Nav1.5 usually shuts down after a few milliseconds. During phase 1 of the AP, the K^+^ channel opens and a large amount of K^+^ is effluxed, causing the primary repolarization of the cardiomyocyte. During phase 2 of the AP, the Ca^2+^ channel opens and Ca^2+^ slowly flows inward while K^+^ continues to flow outward, and the balance between the two causes the membrane potential to change very slowly, forming the plateau phase of the AP. During this plateau phase, the open LTCC on the T-tubule allows external Ca^2+^ to enter the cell. This Ca^2+^ influx not only helps maintain the plateau but also, together with Ca^2+^ already present in the cytoplasm, continues to modulate the RyR2 activity, thereby sustaining the CICR process [56,57]. In phase 3 of the AP, the Ca^2+^ influx ceases and the K^+^ efflux is gradually enhanced, causing the membrane potential to decrease rapidly until it returns to the resting potential level [58,59]. In phase 4 of the AP, when the membrane potential has been stabilized, the voltage-gated ion channels responsible for the AP are basically closed (see Figure 3).

Overall, the storage release of Ca^2+^ mediated by RyR2 is balanced mainly by the reuptake of SERCA2a, whereas the Ca^2+^ influx via LTCC is balanced mainly by the Ca^2+^ extrusion via NCX [60]. Ca^2+^ plays a key role in the contractile and diastolic processes of cardiomyocytes. When cardiomyocytes are stimulated, Ca^2+^ enters the cells and binds to troponin through the above mechanism, triggering the contraction of muscle fibers, while Ca^2+^ is also involved in regulating the contraction strength and velocity of muscle fibers. During diastole, Ca^2+^ is pumped out of the cell, and the muscle fibers relax to prepare for the next contraction [61,62]. Ca^2+^ maintains the equilibrium state inside and outside the cell through the various ion channels mentioned above in order to maintain the steady state of the organism.

## 5. RyR2 Regulation

**Figure 4:**
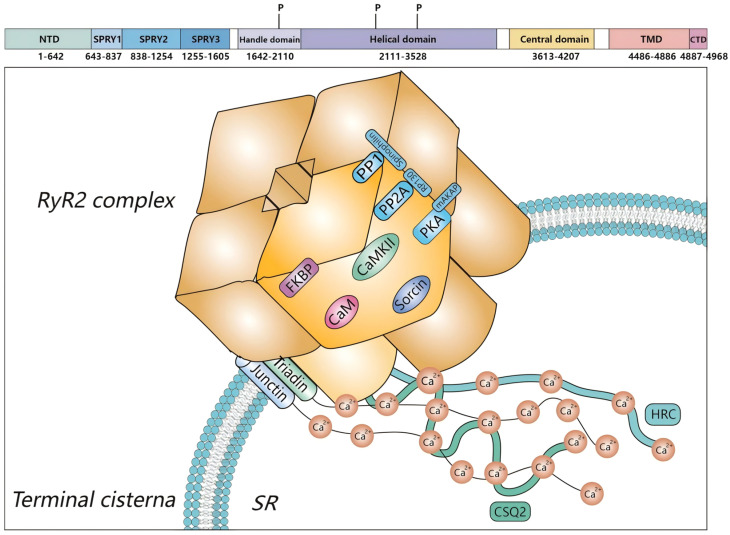
Correlation factors of RyR2 regulation. RyR2, the largest calcium release channel in cardiomyocytes, is a typical tetrameric structure, and its channel activity is not only influenced by itself but also regulated by related factors. The figure shows the common associated factors that bind to RyR2 and thus regulate its activity. P refers to the phosphorylation sites on RyR2.

As the most important Ca^2+^ release channel in cardiomyocytes, RyR2 activity is affected by a number of factors. In addition to mutation in the structure of RyR2, the activity of RyR2 also can be regulated by various modulators, including physiological agents and messengers (such as Ca^2+^ and Mg^2+^), kinases (such as PKA and CaMKII), small regulating proteins (such as CSQ2 and CaM), and various pharmacological agents (such as ryanodine and caffeine) [63,64] (see Figure 4).

### 5.1. RyR2 Mutations: From Molecular Defects to Arrhythmogenic Phenotypes

#### 5.1.1. Cytosolic Ca^2+^ Sensing Mutations

E3987 residing at the central domain of RyR2 is proposed to play a significant role in the cytosolic Ca^2+^ activation of RyR2; the mutation of this key amino acid residue to an alanine, which is unable to interact with Ca^2+^, markedly reduces or even abolishes the activation of RyR2 by cytosolic Ca^2+^. Indeed, the E3987A mutation abolishes the caffeine response and causes a more than 1000-fold decrease in Ca^2+^-dependent [^3^H] ryanodine binding, indicating that the E3987 residue is critical to cytosolic Ca^2+^ activity. The same occurs at site E4032 in RyR1, near site E3885 in RyR3 [65,66,67]. RyR3 mutation E3885A has been shown to form a functional channel without alteration of regulation by other modulators except for a ~10,000-fold decrease in Ca^2+^ sensitivity. The corresponding E4032A mutation in RyR1 is also critical to the channel function as it abolishes the caffeine activity of the channel and Ca^2+^-dependent [^3^H] ryanodine binding [66].

Recent structural studies on the RyR1 have also revealed that the putative cytosolic Ca^2+^ coordination site is between residues E3893 and E3967 from the central domain, and T5001 from the C-terminal domains [21]. The location is close to the E4032 residue which has been shown to be critical in the Ca^2+^-dependent [^3^H] ryanodine binding. It has also been suggested that the E4032 residue forms an interface between the C-terminal domains and permits Ca^2+^ binding. These observations further indicate that the corresponding residue E3987 in RyR2 may play an important role in the regulation of Ca^2+^ binding and the cytosolic Ca^2+^-dependent activation of RyR2. However, numerous other amino acid residues also contribute to fine-tuning RyR2’s response to Ca^2+^. For example, residue 4750, which is a bit away from E3987, is a lysine (K), and, when it is mutated to a glutamine (Q), RyR2 not only becomes more sensitive to cytosolic Ca^2+^, but also becomes completely resistant to inhibition by a high concentration of cytosolic Ca^2+^ [68].

#### 5.1.2. Luminal Ca^2+^-Sensing Mutations

In addition to sensing the concentration of Ca^2+^ in the cytoplasm, RyR2 also constantly monitors the concentration of free Ca^2+^ in the lumen of the SR. Though the SR luminal Ca^2+^ content clearly plays a pivotal role in the function of RyR2, the molecular basis of the luminal Ca^2+^ regulation is not well-understood. Single-channel studies further prove that the cytosolic and luminal Ca^2+^ activation can be specifically regulated [69]. Similarly, the RyR2 mutation E3987A diminishes the cytosolic Ca^2+^ activation of the channel without altering the luminal Ca^2+^ activation [67]. Therefore, the selective effect of Ca^2+^-dependent activation suggests that the luminal Ca^2+^ activation is distinct from the cytosolic Ca^2+^ activation of RyR2. There should be a binding and regulatory site on the luminal side that regulates the function of RyR2.

The putative luminal Ca^2+^-binding site has not been determined. Recent studies revealed a residue E4872 located at the helix bundle crossing is critical for luminal Ca^2+^ regulation. The E4872Q mutation completely abolishes luminal, but not cytosolic, Ca^2+^ activation [70]. However, recent studies on RyR2 3D structure have shown that E4872 is located at the cytosolic side of the RyR2 channel pore, though close to the luminal side. These studies also suggest that E4872 may form an inter-subunit salt bridge with R4874 and contribute to the formation of a cation-binding pocket that is involved in the luminal Ca^2+^ activation of RyR2 [24]. Therefore, it is possible that E4872 regulates the luminal activation of RyR2 by forming salt bridges and facilitating the formation of a Ca^2+^-binding pocket.

Defective luminal Ca^2+^ regulation has been linked to the pathogenesis of various cardiac diseases. As early as 1972, it was demonstrated that an increased SR Ca^2+^ load could lead to propagating Ca^2+^ waves and spontaneous contractions in skinned muscle fibers [71]. This spontaneous Ca^2+^ release event is induced by the overload of the SR Ca^2+^ store and is different from depolarization-triggered CICR. Therefore, it is termed store-overload-induced Ca^2+^ release (SOICR) to differentiate it from the physiological CICR. The resultant severe SR Ca^2+^ spillover can lead to abnormal activity and triggered arrhythmias. For normal RyR2, it is essentially impossible for SOICR to occur in the physiological state. However, the K4750Q mutation can greatly reduce the SOICR threshold of RyR2, leading to CPVT. The degree of RyR2 dysfunction caused by the K4750Q mutation is greater than that caused by other known CPVT-associated RyR2 mutations [72].

#### 5.1.3. Clinical Mutation Clusters: CPVT and Beyond

Almost every region of the RyR2 protein is involved in channel regulation, and the total number of disease-associated RyR2 mutations is more than 300, which can cause various types of arrhythmias, including CPVT [73,74], with the associated RyR2 mutations concentrated in four discrete regions of RyR2 (CPVT-I, 77–466; CPVT-II, 2246–2534; CPVT-III, 3778–4201; and CPVT-IV, 4497–4959) [75]. In skeletal muscle, point mutations in RyR1 cause malignant hyperthermia and central core disease, and, in cardiac muscle, RyR2 mutations cause CPVT and other arrhythmias [76,77,78,79].

Several catecholaminergic polymorphic ventricular tachycardia (CPVT)-associated RyR2 mutations have been shown to enhance luminal Ca^2+^ activation by reducing the SOICR threshold, and promote the genesis of triggered arrhythmias. Similarly, the loss-of-function CSQ2 mutations, which reduce the binding and buffering capacity for luminal Ca^2+^, cause an increase in the SR free Ca^2+^ level and enhance the propensity for SOICR and triggered arrhythmias [2]. These findings suggest that the increased luminal Ca^2+^ activation of RyR2 may be a common defect of cardiac diseases such as CPVT.

#### 5.1.4. Gain-of-Function vs. Loss-of-Function: A Spectrum of Phenotypes

Conversely, a unique RyR2 mutation A4860G associated with idiopathic ventricular fibrillation (IVF) abolishes luminal Ca^2+^ activation, indicating that the decreased luminal Ca^2+^ sensitivity of RyR2 can also cause cardiac disease [80]. This highlights that both gain-of-function (enhanced SOICR, as seen in most CPVT mutations) and loss-of-function (reduced luminal Ca^2+^ sensitivity, as seen in A4860G) mutations in RyR2 can lead to arrhythmogenic phenotypes, underscoring the delicate balance required for proper RyR2 function.

### 5.2. Modulation of RyR2 Activity

#### 5.2.1. Ca^2+^

Since cytosolic Ca^2+^ is the major activator of RyR2 during the process of CICR, the basic mechanism of Ca^2+^ sensing by RyR2 has been extensively studied. It has been shown that, in the absence of Mg^2+^, RyR2 activation exhibits a bell-shaped dependence of Ca^2+^ concentration, suggesting that Ca^2+^ activates and inactivates RyR2 in a concentration-dependent manner. RyR2 is thereby activated by low-cytosolic Ca^2+^ binding to its high-affinity site and inhibited by high-cytosolic Ca^2+^ binding to its low-affinity site. Cytosolic Mg^2+^ is also able to bind to the RyR2 Ca^2+^-binding sites, especially to the low-affinity inhibitory site, and inhibits the channel [81,82,83,84]. The inhibition of RyR2 by Mg^2+^ can be markedly relieved by elevating cytosolic Ca^2+^. During ECC, the physiological range of cytosolic Ca^2+^ can fully open the RyR2 channels and cause a subsequent SR Ca^2+^ release.

The level of the SR Ca^2+^ store is maintained by the balance between the Ca^2+^ uptake via SERCA2a and the Ca^2+^ release via RyR2. The intra-SR Ca^2+^-buffering protein CSQ2 allows the SR to store up to ~20 mM Ca^2+^ while the free SR Ca^2+^ level remains at ~1 mM. The presence of bound Ca^2+^ enables the efficient storage and release of a large amount of Ca^2+^ within the relatively small SR luminal space (3.5% of total cell volume) [3].

The physiological role of luminal Ca^2+^ has been substantially characterized. In addition to the role in Ca^2+^ release termination, luminal Ca^2+^ may also be a key player in SR Ca^2+^ release activation. Single-channel recordings in artificial lipid bilayers from different groups consistently show that luminal Ca^2+^ directly regulates the activity of RyR2. An increased luminal Ca^2+^ level enhances the RyR2 activity while a decreased luminal Ca^2+^ level leads to a reduction in RyR2 activity [69,85,86]. A growing body of evidence suggests that the increased SR Ca^2+^ level promotes RyR2 function and SR Ca^2+^ release while a decreased SR Ca^2+^ content inhibits hte SR Ca^2+^ release in cardiomyocytes [87,88,89,90].

#### 5.2.2. FKBP12.6 (Calstabin 2)

The FKBP family refers to a group of immunoglobulins that bind the immunosuppressant FK506. They are characterized by their ability to bind FK506. FKBP12 and FKBP12.6, members of this family, bind with a high affinity in both the open and closed states of the RyRs, thereby helping to stabilize the channels [84,91]. FKBP12 primarily regulates RyR1, whereas FKBP12.6 regulates RyR2 [92,93]. FKBP12 and FKBP12.6 have been shown to stabilize the closed state of the channel, preventing Ca^2+^ leakage from the SR, and, when FKBP12.6 is separated from RyR2, it leads to Ca^2+^ leakage from the SR [94].

FKBP12.6 was one of the first RyRs regulators to be discovered, and can bind to all three RyRs isoforms [95], with the highest affinity for RyR2, but, because it is not expressed at a very high level in myocardial tissues, only about 10–20% of RyR2 binds to it [96,97]. The binding of FKBP12.6 puts RyR2 in an inhibited state, and it has been shown that the binding site of FKBP12.6 on RyR2 is the same as that of FKBP12 on RyR1 and is located in the gap formed by the SPRY1, SPRY3, NTD, and handle structural domains [29,30]. FKBP12.6 stabilizes RyR2 in its closed state by relaxing the central structural domains, even though, in the case of Ca^2+^ with PCB95, Ca^2+^ with ATP, or Ca^2+^ with caffeine, they are not sufficient to open the channel. However, the combined action of caffeine and ATP in the presence of Ca^2+^ can open the channel in the presence of FKBP12.6 [98]. These findings support a role for FKBP12.6 in the pathophysiological regulation of RyR2 [94,99].

FKBP12/12.6 binding stabilizes RyRs inter-subunit interactions, a finding initially demonstrated by bilayer experiments in the last century [100]. These effects can be reversed by FK506, which binds to FKBP12/12.6 at the RyRs-binding site and inhibits the interaction [101]. Furthermore, FKBP12/12.6 plays an important role for the functional rather than physical coupling of RyRs. Coupling gating provides a mechanism to simultaneously close all RyR2 channels within a T-tubule/SR junction, thereby reducing the likelihood that an individual RyR2 channel will be reactivated by Ca^2+^ through neighboring channels [40,102].

The binding of FKBP12/12.6 is essential for the stabilization of RyRs, which has been verified in subsequent studies, but is also partly controversial. First, in the knockout model, different strains of FKBP12.6 knockout mice may exhibit different characteristics; some mice have a normal cardiac structure and normal electrocardiograms and show arrhythmias only during stress; some mice do not have exercise-induced arrhythmias but show male-specific cardiac hypertrophy; and others have no obvious changes or any tendency to develop arrhythmias, and their myocardial RyR2 function also showed a normal state [103,104,105].

Second, the frequency, amplitude, and duration of diastolic calcium sparks were reduced after the overexpression of FKBP12.6 in rabbit or mouse myocardium, suggesting an inhibition of the spontaneous opening of RyR2, which has been interpreted as a decrease in diastolic Ca^2+^ leakage leading to an increase in Ca^2+^ stores in the SR [106,107]. Consequently, the amplitude of systolic Ca^2+^ transients and the resulting contractile force were enhanced, likely due to the elevated SR Ca^2+^ content [108,109,110]. The researchers also found that FKBP12.6-overexpressing mice were less susceptible to arrhythmias induced by isoproterenol injection combined with rapid pacing, which seems to be attributable to the stabilizing effect of FKBP12.6 on RyR2 [111,112]. However, LTCC and NCX were also affected by FKBP12.6 overexpression, with the former showing a 15% decrease in the peak current density and the latter showing an 18% decrease in protein levels [60,112,113]. These additional effects could also influence arrhythmogenesis, complicating the interpretation.

In conclusion, although most of the evidence supports a stabilizing effect of FKBP12.6 on RyR2, the importance of FKBP12.6 regulation is still quite controversial. How FKBP12.6 dysfunction further induces arrhythmias after it impairs the Ca^2+^ release function of RyR2 in cardiomyocytes and whether it involves the modulation of other key factors are questions warranting in-depth investigation.

#### 5.2.3. CaM (Calmodulin)

CaM is a major modulator of a wide range of cellular processes [114]. It is a 16.7 kDa cytosolic protein with four EF-hand Ca^2+^-binding sites that form two symmetrical domains (the N- and C-domain) connected by a flexible α-helix. The N-domain contains two low-affinity Ca^2+^-binding sites (K_d_~12 µM) while the C-domain contains two high-affinity Ca^2+^-binding sites (K_d_~1 µM) [115,116]. The binding of Ca^2+^ alters the interhelical angle in the EF-hand motif, resulting in a conformational change that exposes the hydrophobic binding site and allows CaM to wrap around its target proteins.

In cardiomyocytes, CaM affects the cardiac ECC by modulating the function of a variety of ion channels, including LTCC, IP3R, and RyR2. CaM regulates RyR2 not only through the CaM–CaMKII pathway but also by direct interaction between CaM and RyR2. CaM is able to bind to RyR2 in both the Ca^2+^–CaM (Ca^2+^-bound form of CaM) and apo-CaM (Ca^2+^-unbound form of CaM) forms and inhibit RyR2 activity [117]. With saturating cytosolic Ca^2+^, both the N- and C-domains bind to RyR2, while the C-domain can bind RyR2 at resting cytosolic Ca^2+^ [118,119,120,121].

Therefore, it is speculated that the C-domain of CaM is mainly responsible for the inhibition of RyR2 at low-cytosolic Ca^2+^, while both the N- and C-domains can sense the elevated Ca^2+^ level and increase the inhibitory effect on RyR2. Single-channel studies have demonstrated that CaM effectively reduces the open probability at a Ca^2+^ concentration lower than 10 µM, while, at a Ca^2+^ concentration higher than 10 µM, the inhibition of CaM is less efficient. This result suggests that the inhibitory effect of CaM is insignificant during the active phase of SR Ca^2+^ release due to the high local cytosolic Ca^2+^. As soon as the Ca^2+^ level drops to a certain range, CaM initiates the inhibition on RyR2 and helps with the termination of SR Ca^2+^ release [122].

CaM binds to RyR2 channels with a maximum ratio of four per channel via the RyR2 CaM-binding domain (Arg3581–Pro3607) [123,124,125]. This domain is located at the boundary of three domains according to the recently resolved RyR2 3D structure. Recently, an interesting structural study found that CaM bound to the third CaM-binding site of RyR2 can bind the fifth Ca^2+^, and the extra Ca^2+^ is not in the EF-hand motif but in the linkage region in the middle of the two structural domains of calmodulin [126]. This finding may have implications for CaM binding to RyR2, although the observation lacks substantial experimental validation and requires further investigation.

Recently, several CaM mutations linked to a variety of cardiac arrhythmias such as CPVT, long QT syndrome (LQTS), and IVF have been shown to alter the function of RyR2 [127,128,129]. The location of these mutations suggests that the mechanism of the resultant alterations is complex. Some mutations located at the EF-hand motif have been shown to alter the properties of Ca^2+^ binding and therefore affect the function of RyR2 [130]. Engineered CaM variants that abolish Ca^2+^ binding also lead to an altered RyR2-mediated SR Ca^2+^ release [131]. In cardiomyocytes, almost all of the arrhythmogenic CaM mutations identified are located in the CaM C-terminal structural domain and have a significant impact on the interaction of RyR2 with CaM [132]. In recent years, our studies have revealed that Ca^2+^–CaM-dependent RyR2 inactivation plays an important role in intact-cardiac-pacing-induced Ca^2+^ turnover and developed a new numerical cardiomyocyte model of Ca^2+^ turnover for validation, finding that the pacing-induced elevation of cytoplasmic Ca^2+^ in diastole drove the Ca^2+^–CaM-dependent inactivation of RyR2, which, when sufficiently high, resulted in an SR Ca^2+^ release–uptake imbalance [133].

There is now growing evidence that CaM is an important RyR2-stabilizing factor, comparable in this role to FKBP12.6. The abnormal dissociation of FKBP12.6 is thought to play a role in many acquired or congenital pathological processes, a claim that also applies to CaM and is supported by much evidence. Most of the disease-associated CaM mutations studied to date appear to reduce the inhibitory effect of CaM on RyR2 [134,135,136], and aberrant interactions between CaM and RyR2 have been associated with heart failure [137,138,139]. Correcting these impaired CaM–RyR2 interactions may represent a therapeutic strategy for treating pressure-overload-induced lethal arrhythmias in failure hearts [140,141]. However, the exact pathogenic mechanism of each type of arrhythmia and the effects of disease-associated CaM mutations on the RyR2 function remain to be determined.

S100A1 is a Ca^2+^-binding protein with a molecular weight of 21 kDa that also binds to the CaM-binding site of RyR2 to exert regulatory effects [142,143,144]. Competition binding experiments have shown that S100A1 shares a common binding site with CaM on RyR1 [145]. However, mutation and functional experiments indicated that S100A1 and CaM do not share identical binding sites in RyR2 [138,146]. Recent FRET experiments suggested that S100A1 interacts allosterically with the CaM-binding sites in RyR1 and RyR2, rather than through direct competitive binding [147]. It has been claimed that skeletal muscle lacking S100A1 exhibits a Ca^2+^ transient peak and that S100A1 protein expression is decreased in the presence of heart failure [148], and that increasing its expression significantly increases myocardial contractility, thereby inhibiting heart failure [149].

#### 5.2.4. CSQ2 (Calsequestrin 2), Triadin, Junctin, and HRC

These four proteins are all present in the SR, of which Triadin and Junctin are single transmembrane proteins on the SR membrane with similar structures, all of which extend their long C-termini into the SR lumen to provide binding sites for regulatory factors including CSQ2 [150], of which Triadin is involved in the formation and maintenance of the dyadic cleft structure [151], and Junctin plays a role mainly through the direct regulation of RyR2 [152]. CSQ2 and HRC are calcium-buffering proteins located in the SR and are essential for the ECC [54].

The two isoforms of the CSQ family, CSQ1 and CSQ2, are predominantly expressed in skeletal and cardiac muscle, respectively, and, although they do not have a high affinity for calcium, they have a high capacity, binding 40–50 calcium ions and accounting for the majority of Ca^2+^ buffering in the SR [153]. When the surrounding Ca^2+^ concentration is high, CSQ2 binds Ca^2+^; when it is low, CSQ2 rapidly releases bound Ca^2+^, thereby buffering [Ca^2+^]_i_ to about 1 mM. During each Ca^2+^ transient, only 35–40% of the SR Ca^2+^ store is released without causing store depletion, a property largely attributable to CSQ2 [154,155].

In addition to buffering Ca^2+^, an important role of CSQ2 is its regulatory effect on RyR2, which is overall inhibitory. It may regulate RyR2 through two mechanisms: one involves directly binding to RyR2 and thereby exerting a regulatory effect, while the other involves indirect regulation via interactions with other RyR2-binding proteins. The direct action is through the interaction of the C-terminal tail with the first SR luminal loop of the transmembrane portion of RyR2 [153]; the indirect action is relatively complex and has been studied in greater depth. A series of studies have demonstrated that CSQ2 is linked to RyR2 through Triadin and Junctin proteins on the SR membrane [156,157,158], and that Triadin and Junctin anchor CSQ2 to the connecting SR through charge interactions [151,159].

The two SR membrane proteins, Triadin and Junctin, bind to different sites in the RyRs, but both promote calcium release through the RyRs [160]. Triadin is mainly involved in the maintenance of the microstructure of the dyadic cleft and the expression of other calcium-regulated proteins in the relevant tissues, whereas Junctin regulates the activity of RyR2 in a more direct manner, but little is known about its specific physiological role [161]. In contrast, CSQ2 mainly inhibits RyRs [162,163], but the binding of CSQ2 to RyR2 is also closely related to the concentration of calcium in the SR lumen, and it has been suggested that CSQ2 can act as a calcium receptor for RyR2 in the SR lumen [164]. When the calcium concentration increases gradually, CSQ2 binds to Triadin and Junctin to form a ternary complex, which is anchored to the SR and inhibits the opening of the RyR2 channel, and this inhibitory effect decreases with the increase in calcium concentration [165]; when the calcium concentration exceeds 5 mM, the ternary complex gradually dissociated and the inhibitory effect of CSQ2 was further reduced [158].

Triadin and Junctin bind not only to CSQ2 and RyR2, but also to HRC via the KEKE sequence at the C-terminus [151,152]. As mentioned earlier in the chapter on Ca^2+^ homeostasis, HRC is also a calcium-buffering protein located in the SR, named for its histidine-rich content, and is a novel regulator of SR Ca^2+^ uptake, storage, and release. The effect of HRC on RyRs may be modulated by its Ca^2+^ sensitivity to interact with Triadin. A related study found that HRC overexpression was accompanied by elevated levels of Triadin and Junctin, while RyRs, CSQ, PLN, and SERCA remained unchanged [166], increased the SR Ca^2+^ storage capacity [167], and had an even more pronounced effect on calcium transients and cardiomyocyte contractility than the 20-fold overexpression of CSQ [168]. There are relatively few studies on HRC, and a large amount of experimental data is still needed for future confirmation.

The ability of CSQ2 to rapidly bind and release Ca^2+^ is essential to provide sufficient Ca^2+^ for force generation, and, in addition, the normal function of CSQ2 is critical to prevent spontaneous RyR2 Ca^2+^ release and the development of arrhythmias. It has been found that CSQ2 knockout mice exhibit stress arrhythmias and are highly susceptible to CPVT [169,170], and CPVT-associated CSQ2 mutations may lead to impaired Ca^2+^ buffering, multimer formation, and RyR2 regulation [45].

#### 5.2.5. DHPR (Dihydropyridine Receptor)

DHPR is a type of LTCC, specifically a member of the Cav1 family (Cav1.1, Cav1.2, and Cav1.3). It is a tetramer consisting of four subunits, α1, α2/δ, β, and γ. The primary role of DHPR is to regulate the excitability and contractility of muscle cells, and it is expressed in both skeletal and cardiac muscle [171]. DHPR is known to be a regulator of RyR function, and the case of Cav1.1 acting on RyR1 belongs to the direct mechanical interaction, and the case of Cav1.2 acting on RyR2 belongs to the indirect CICR release pathway [172,173]. By mediating a small influx of extracellular Ca^2+^, DHPR triggers the opening of RyR2 channels on the SR membrane, leading to a large-scale Ca^2+^ release. Thus, DHPR is a major trigger for RyR2 release in cardiomyocytes and plays a crucial role in myocardial ECC.

In skeletal muscle, the SPRY2 structural domain at the N-terminal of RyR1 is thought to interact with Cav1.1 [174], and RyR1 is regulated by DHPR; therefore, DHPR abnormalities are directly related to RyR1 function [175,176], and mutations in DHPR can cause malignant hyperthermia [177]. Later on, another researcher found a significant co-localization of Cav1.3 with RyR2 in rat hippocampal neurons [178], and high-resolution cryo-electron microscopy experiments also demonstrated a functional coupling between the RyR2–Cav1.3 cluster and intracellular Ca^2+^ levels and SR Ca^2+^ [179]. Despite the high-resolution structural resolution of RyR1/2 and DHPR [180,181], the supercomplex between the two channels has not been reconstructed in vitro. However, a recent study showed that, in tsA201 cells, the co-expression of Cav1.1, β1a, Stac3, RyR1, and junctophilin2 induced the formation of junctions between the SR membrane and plasma membrane, suggesting that these proteins are essential for ECC [182].

#### 5.2.6. Sorcin

Sorcin is a 22-kDa calcium-binding protein that is widely found in a variety of tissues, most abundantly in cardiac myocytes, where it binds directly to the head of RyR2 and thereby exerts a regulatory effect. Sorcin was initially identified in fibroblast cell lines resistant to a variety of chemotherapeutic agents, and it was later found to interact with both RyR2 and LTCC, with the overall effect being to inhibit the action of CICR [183]. It exerts a strong inhibitory effect on RyR2, even reducing the probability of the channel’s steady state opening to zero at higher concentrations, and this inhibitory effect is not very dependent on calcium concentration, as shown by some in vitro experiments, and reduces the “gain” of the ECC in general [184,185,186]. It is worth mentioning that Sorcin can also be phosphorylated by PKA, significantly reducing the inhibitory effect on RyR2 [187].

In recent years, in order to study the effects of Sorcin on cardiac ECC and its role in the development of cardiac dysfunction, some researchers have conducted experiments using the Sorcin–KO mouse model and found that Sorcin deficiency may trigger ventricular arrhythmias due to Ca^2+^ interference, which demonstrates the critical role of Sorcin in maintaining Ca^2+^ homeostasis, especially during the adrenergic response of the heart [188]. It has also been shown that Sorcin is associated with neurodegenerative diseases, as Sorcin counteracts the elevated cytoplasmic calcium levels associated with neurodegenerative diseases, modulates endoplasmic reticulum Ca^2+^ transients, and even increases the rate of SR calcium uptake by increasing the SERCA activity [189].

#### 5.2.7. Phosphorylation/Dephosphorylation (PKA, CaMKII, PP1/2A, and PDE4D3)

Protein kinase A (PKA) is a key player in the β-adrenergic-signaling pathway. The stimulation of the β-adrenergic receptor activates adenylyl cyclase and increases the level of cAMP, which, in turn, activates PKA [3,190]. There are several major protein targets in cardiomyocytes for PKA, including the LTCC, RyR2, and PLB. The phosphorylation of these proteins leads to an increased Ca^2+^ influx, enhanced SR Ca^2+^ release, and increased contractility and cardiac output [191,192].

After RyR2 is phosphorylated by PKA, RyR2 will be further activated, which is manifested at the single-channel level by the increased conductance, increased open probability, and enhanced sensitivity of RyR2 to cytosolic Ca^2+^. During the ECC process, phosphorylation increases the frequency, size, and degree of synchronization of diastolic calcium sparks, enhances the fractional Ca^2+^ release (the proportion of Ca^2+^ released from the SR as a percentage of the original amount of SR Ca^2+^), and increases the coupling fidelity of RyR2 [193,194]. However, the above results are partly controversial. For example, some people did not observe a significant effect of PKA on ECC [195], while others reported that the Ca^2+^ release fraction remained unchanged or even decreased after PKA activation [196]. Some single-channel experiments have observed that RyR2 phosphorylated by PKA becomes more sensitive. This means the frequency of opening is increased, and it is more sensitive to cytosolic Ca^2+^ activation, which releases more Ca^2+^, but it closes faster after each opening; as a result, it reduces the stability of the channel [197].

Two phosphorylation sites on RyR2, S2030 and S2808, have been identified and widely accepted [190,198]. Additional functional studies revealed that the PKA phosphorylation of RyR2 enhances luminal Ca^2+^ activation and reduces the SOICR threshold [198]. As the residue S2808 is substantially phosphorylated under basal conditions, the functional effect of PKA activation is mainly dependent on the phosphorylation of S2030. In a failing heart, the residue S2808, which is also a Ca^2+^/CaM-dependent protein kinase II (CaMKII) site, is hyperphosphorylated under basal conditions [190]. However, the basal phosphorylation of S2030 is not enhanced in failing rat hearts and could be markedly increased upon β-adrenergic stimulation [198].

There have also been single-channel studies of RyR2 channel gating that have shown that PKA phosphorylation can overcome the blocking effect of Mg^2+^, allowing the channel to be active at physiological Mg^2+^ concentrations (~1 mM) [199]. There is evidence that FKBP12.6 dissociates more readily from hyperphosphorylated RyR2, and this may play a role in the development of certain cardiac diseases [200].

**Figure 5:**
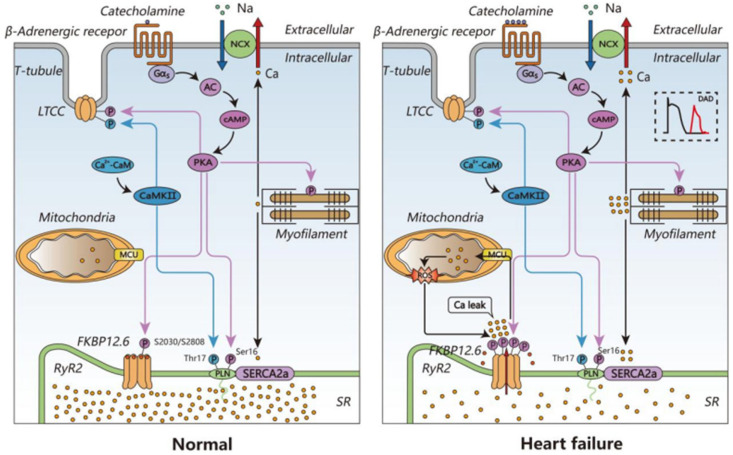
Schematic representation of diastolic intracellular Ca^2+^ handling in normal and failing ventricular cardiomyocytes. In ventricular cardiomyocytes from normal hearts, diastolic Ca^2+^ from the SR via RyR2 is minimal or absent. In contrast, the SR of ventricular cardiomyocytes from failing hearts releases large amounts of diastolic Ca^2+^ via RyR2, and, thus, Ca^2+^ stores in the SR are reduced. In heart failure, increased levels of catecholamines promote phosphorylation of RyR2 and LTCC through a series of pathways that increase level of the cAMP-dependent protein kinase PKA. The pathological leak of Ca^2+^ from the SR is caused by remodeling of RyR2 as a result of hyperphosphorylation by PKA, oxidation, S-nitrosylation, and dissociation of FKBP12.6 from the RyR2 channel complex, resulting in channels that do not close properly during diastole. The increased cytoplasmic concentration of Ca^2+^ can stimulate CAMKII, which phosphorylates LTCC and PLN at Thr17. The cytoplasmic Ca^2+^ is, to some extent, buffered by mitochondria, which produce ROS that can further contribute to RyR2 channel oxidation. Ca^2+^ is removed from the cytoplasm by the SERCA2a, which pumps Ca^2+^ back into the SR, and the NCX, which extrudes Ca^2+^ out of the cell.

Interestingly, the dephosphorylation of the RyR2 S2808 site also induces a modest increase in the RyR2 opening probability and conductance under pathological conditions of HF [201]. However, because PKA can phosphorylate RyR2 at both the S2808 and S2030 sites, the PKA-dependent phosphorylation of S2030 may be specifically responsible for the observed functional effects on individual RyR2 channels [202]. Our recent finding that the RyR2 S2030 phosphorylation site controls Ca^2+^ release termination and Ca^2+^ turnover further provides a link between RyR2 phosphorylation and cardiac diseases such as arrhythmias [203]. These observations suggest that the increased basal PKA phosphorylation of S2808 is a defect in heart failure. Moreover, the altered Ca^2+^ handling to myofilaments due to S2030 phosphorylation could further contribute to cardiac arrhythmias in failing hearts [198] (see Figure 5).

In addition to PKA, CaMKII**_δc_**, which is the predominant isoform expressed in the heart, also phosphorylates and regulates the function of several target proteins. Upon the elevation of cytosolic Ca^2+^, Ca^2+^ binds to CaM, and the resultant Ca^2+^–CaM complex binds to the regulatory site of CaMKII and activates it [204,205]. The activated CaMKII phosphorylates the LTCC, RyR2, and PLB, and generates various functional alterations. CaMKII exhibits a dual role in its physiological effects on RyR2, with single-channel studies reporting that it either increases or decreases the open probability of RyR2, the latter also protecting RyR2 from inhibition by Mg^2+^ [206,207,208]. CaMKII also phosphorylates other targets that are not directly related to Ca^2+^ handling, such as Na^+^ and K^+^ channels, and regulates their functions [209,210].

CaMKII influences ECC processes by phosphorylating RyR2 [206,211,212]. Cellular experiments have shown that CaMKII activity increases the diastolic Ca^2+^ leak. Conversely, reducing the CaMKII activity with specific inhibitory peptides decreases both the systolic SR Ca^2+^ release and the frequency of diastolic Ca^2+^ sparks [213,214]. Constitutively active CaMKII has been shown to inhibit RyR2 activity, reduce Ca^2+^ sparks, and decrease Ca^2+^ transients. Researchers hypothesized that this phenomenon may be a compensation for the over-activation of CaMKII, which is used to prevent the loss of ECC control [215,216].

**Figure 6:**
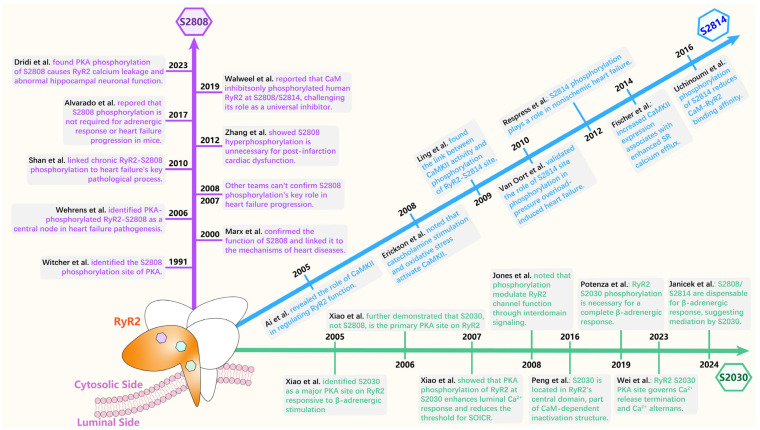
Timeline of key discoveries and controversies in RyR2 phosphorylation. This figure summarizes the major phosphorylation sites identified on RyR2 and illustrates the evolution of the debate regarding their functional roles in cardiac physiology and disease [190,222,223,224,225,226,227,228,229,230,231,232,233,234,235,236,237,238,239,240,241].

CaMKII does not have only one phosphorylation site; aside from S2808, S2814 is another identified CaMKII site of RyR2. Numerous studies have shown that the abnormal phosphorylation of RyR2 due to the altered CaMKII_δc_ expression and activity contributes to the pathogenesis of cardiac disease conditions including HF and cardiac arrhythmias [217]. For instance, a transgenic CaMKII mouse develops HF, whereas mice overexpressing the CaMKII inhibitor or with deficient CaMKII are protected from developing HF [218,219,220]. Further, S2814D mutant mice mimicking constitutively phosphorylated S2814 are susceptible to ventricular tachycardia (VT) without changes in the heart structure [221]. However, even after decades of studies, the pathogenic mechanism of the abnormal CaMKII phosphorylation of RyR2 remains controversial [190,208,222,223,224,225,226,227,228,229,230,231,232,233,234,235,236,237,238,239,240,241], which needs to be further investigated in the future (see Figure 6).

In summary, significant research on RyR2 phosphorylation has established that both PKA and CaMKII phosphorylate distinct sites on the channel, with PKA targeting S2030 and S2808, and CaMKII targeting S2808 and S2814, leading to functional alterations such as increased channel activity, enhanced Ca^2+^ release, and reduced stability, which are critical for cardiac contractility and excitation–contraction coupling. However, controversies persist, including conflicting reports on whether PKA phosphorylation increases or decreases the fractional Ca^2+^ release, variability in the single-channel observations regarding RyR2 sensitivity and gating kinetics, and unresolved debates over the pathogenic mechanisms of CaMKII-mediated phosphorylation in heart failure and arrhythmias, particularly regarding whether increased phosphorylation or compensatory dephosphorylation drives disease progression.

In addition to RyR2, PKA and CaMKII can also phosphorylate LTCC and PLN. The current is enhanced by LTCC phosphorylation [242], and PLN phosphorylation loses the ability to inhibit SERCA2a, improves the efficiency of SR Ca^2+^ loading, accommodates higher-frequency AP and increases the ability of the SR to store Ca^2+^, which enhances the contractile force [40].

Since there is phosphorylation, there must be dephosphorylation, mainly by phosphatases and phosphodiesterases, specifically PP1/2A and PDE4D3 [243]. Interestingly, PP1 itself is also the target of PKA phosphorylation, and is inhibited by phosphorylation. To be uninhibited, PP1 needs to be dephosphorylated by PP2A [244]. In summary, it is a complex physiological process. PDE4D3 terminates PKA signaling by degrading cAMP. The inhibition of PDE4D3 would therefore lead to elevated cAMP levels and prolonged PKA activation. There are two phosphorylation sites on PDE4D3 (serine 13 and 54), and these two sites are targets of PKA. After PKA phosphorylates PDE4D3, its activity is enhanced, which, in turn, hydrolyzes cAMP near PKA faster, and, as a result, PKA is, in turn, inhibited, acting as a negative feedback loop. In addition, PP1 and PP2A also affect SERCA activity through PLB dephosphorylation [245].

#### 5.2.8. ROS (Reactive Oxygen Species)

During RyR2 activation, the local transfer of Ca^2+^ to the mitochondrial matrix activates enzymes in the tricarboxylic acid cycle and drives the electron transport chain (ETC) to accelerate ATP production [246], thereby providing a link between the Ca^2+^-dependent contraction and mitochondrial metabolic output. It is well-established that, in cardiac diseases such as heart failure (HF), an abnormal mitochondrial function is often accompanied by an increased emission of reactive oxygen species (ROS) [247]. Excessive mitochondria-derived ROS (mito-ROS) evoke profound changes in intracellular Ca^2+^ homeostasis [248]. Importantly, a mito-ROS-mediated increase in the activity of RyR2 has been linked to the increased propensity for an aberrant Ca^2+^ leak from the sarcoplasmic reticulum (SR), leading to diminished systolic Ca^2+^ transients and an increased incidence of pro-arrhythmic diastolic Ca^2+^ waves [249].

The impact of ROS on RyR2 function is central to the pathological cycle in cardiac disease states. Excessive mito-ROS can directly oxidize critical cysteine residues on RyR2. This oxidation alters the channel’s conformation, stabilizing a sub-conductance state with an increased open probability and a diminished association with stabilizing subunits such as FKBP12.6. Consequently, the oxidized RyR2 channel becomes hyperactive and leaky, leading to an aberrant diastolic Ca^2+^ release from the SR [247]. This pathological leak depletes the SR Ca^2+^ stores and elevates cytosolic Ca^2+^, promoting delayed afterdepolarizations (DADs) and triggered arrhythmias, as seen in CPVT and HF [250,251].

The enhanced RyR2 activity increases the SR–mitochondrial Ca^2+^ transfer, which, under pathological conditions, can disturb the mitochondrial membrane potential and further stimulate the mito-ROS emission via ETC. This new wave of ROS then oxidizes additional RyR2 channels, creating a positive feedback loop that drives disease progression. Worse still, sustained high levels of ROS contribute to the opening of the mitochondrial permeability transition pore (mPTP), committing the cell to apoptosis, thereby linking acute arrhythmogenic triggers to chronic myocardial remodeling and heart failure deterioration [233]. Therefore, ROS act not merely as a byproduct but as a critical pathological effector that directly modifies RyR2 function and disrupts Ca^2+^ homeostasis.

## 6. Conclusions

**Figure 7:**
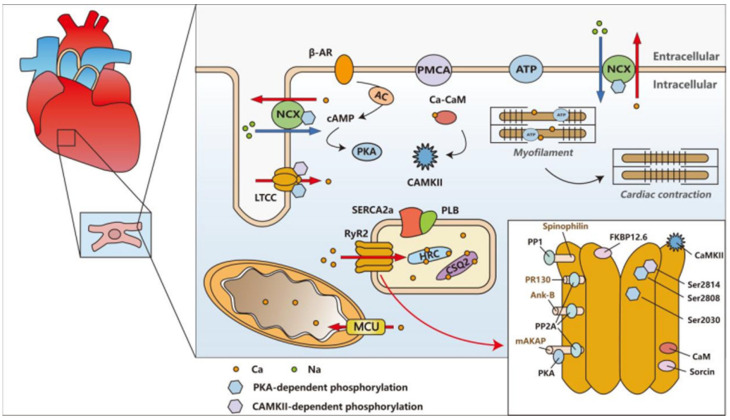
Schematic diagram of cardiac calcium regulation; the lower right corner shows the relevant regulatory sites on RyR2. This integrative illustration summarizes the key molecular components involved in cardiac Ca^2+^ handling, with emphasis on RyR2 regulation and its central role in ECC. AC, adenylate cyclase. cAMP, cyclic adenosine monophosphate. CaM, calmodulin. CaMKII, Ca^2+^/calmodulin-dependent protein kinase II. CSQ2, calsequestrin 2. HRC, histidine-rich calcium binding protein. LTCC, L-type Ca^2+^ channel. NCX, Na^+^/Ca^2+^ exchanger. PKA, protein kinase A. PLB, phospholamban. PMCA, plasma membrane Ca^2+^-ATPase. PP1/2A, protein phosphatase 1/2A. SERCA2a, sarco/endoplasmic reticulum Ca^2+^-ATPase 2a.

There is no doubt that Ca^2+^ has a very important role in the heart, and, over the past 20 years, many studies have demonstrated that Ca^2+^ in cardiomyocytes acts mainly by affecting RyR2; as the largest Ca^2+^ release channel in the cardiomyocyte, it is regarded as a sensor of myocardial function. RyRs have been studied for more than 40 years, and the study of their structure first originated from rabbit RyR1, but the resolution was not very high. After revolutionizing the cryo-electron microscopy technique in 2015, the atomic structure of RyRs was gradually unraveled, and the molecular mechanism of RyR2’s interaction with other key regulators was also understood at the atomic level, and a big step toward resolving the function of RyR2 has been taken since then. In this review, we systematically elucidated how RyR2 is regulated by mutations in RyR2 itself as well as by various key regulators, including FKBP12.6, CaM, and so on (see Figure 7).

If RyR2 is pathologically altered, it can cause a series of cardiomyopathies, including arrhythmias, HF, CPVT, and Ca^2+^ release deficiency syndrome (CRDS) [252,253], which can seriously affect the life and health of the organism. For the time being, there are still many mutations whose effects on the structure of RyR2 remain enigmatic, hindering the rational design of new structure-based therapies. Therefore, a comprehensive and systematic analysis of the mechanisms involved in the RyR2 calcium release channel is crucial, and could serve as a key foundation for new therapies that break through traditional therapies in the future, and will also provide more clues to the challenges in this field.

Looking forward, a comprehensive understanding of the RyR2-mediated cardiac pathophysiology requires positioning this channel within a broader multi-scale framework that encompasses intercellular communication, organelle crosstalk, systemic regulation, and tissue-level repair strategies.

Myocardial infarction creates a heterogeneous electrophysiological environment where border-zone cardiomyocytes harboring dysfunctional RyR2 are particularly vulnerable to mechanical and oxidative stress. Emerging bioengineering approaches, such as self-locking conductive cardiac patches with barbed microneedles, offer a promising strategy to re-establish the electrical continuity across infarcted regions [254]. These patches not only provide an immediate electrical integration but also modulate the calcium-handling-related gene expression, suggesting a potential synergy with RyR2-targeted therapies in restoring synchrony to the failing heart.

Cardiac fibrosis, a key driver of adverse remodeling, is regulated by signaling pathways that may crosstalk with RyR2 complexes. Xanthohumol has been shown to inhibit TGF-β1-induced cardiac fibroblast activation via the PTEN/Akt/mTOR signaling pathway, suppressing proliferation, differentiation, and collagen overproduction [255]. Given that fibrotic tissue alters the mechanical and electrical load on surviving cardiomyocytes, understanding how these pathways intersect with RyR2 stability and function could reveal new nodes for therapeutic intervention.

Mitophagy plays a dual role in myocardial ischemia/reperfusion injury (MIRI). The Pink1/Parkin pathway and receptor-mediated pathways involving FUNDC1 and BNIP3 maintain mitochondrial homeostasis by removing defective mitochondria [256]. However, excessive mitophagy can exacerbate injury by causing uncontrolled mitochondrial depletion [256]. This is particularly relevant to RyR2 function, as mito-ROS production contributes to RyR2 oxidation and a pathological Ca^2+^ leak, while an RyR2-mediated cytosolic Ca^2+^ overload can trigger the mitochondrial Ca^2+^ uptake and permeability transition, establishing a vicious cycle of organelle dysfunction. Therapeutic strategies that fine-tune mitophagy may therefore indirectly protect RyR2 from oxidative damage.

At the systemic level, hormonal regulation adds another layer of complexity to RyR2 pathophysiology. The G-protein-coupled estrogen receptor (GPER) has emerged as an important mediator of cardiovascular protection, with the emerging evidence implicating its role in modulating Ca^2+^ handling and arrhythmia susceptibility [257]. Understanding how GPER signaling interfaces with RyR2 regulatory networks may provide insights into sex-specific differences in heart disease and open new avenues for pharmacotherapeutic intervention.

In conclusion, while significant progress has been made in elucidating the structural and molecular basis of RyR2 regulation, translating these insights into effective therapies will require an integrated approach that considers the channel’s role within the broader ecosystem of the failing heart. Bridging the gap between molecular defects, cellular responses, tissue-level remodeling, and systemic modifiers represents both a formidable challenge and an exciting frontier for future research.

## Data Availability

No new data were created or analyzed in this study.

## Abbreviations

The following abbreviations are used in this manuscript:

AC	Adenylate cyclase
AP	Action potential
cAMP	Cyclic adenosine monophosphate
CaM	Calmodulin
CaMKII	Ca^2+^/calmodulin-dependent protein kinase II
CSQ2	Calsequestrin 2
CICR	Ca^2+^-induced Ca^2+^ release
CPVT	Catecholaminergic polymorphic ventricular tachycardia
CRDS	Ca^2+^ release deficiency syndrome
DAD	Delayed afterdepolarization
DHPR	Dihydropyridine receptor
ECC	Excitation–contraction coupling
ETC	Electron transport chain
GPER	G-protein-coupled estrogen receptor
HF	Heart failure
HRC	Histidine-rich calcium binding protein
IP3R	Inositol 1,4,5-trisphosphate receptor
LTCC	L-type Ca^2+^ channel
MIRI	Myocardial ischemia/reperfusion injury
mPTP	Mitochondrial permeability transition pore
NCX	Na^+^/Ca^2+^ exchanger
NKA	Na^+^/K^+^-ATPase
PDE4D3	Phosphodiesterase 4D3
PKA	Protein kinase A
PLB	Phospholamban
PMCA	Plasma membrane Ca^2+^-ATPase
PP1/2A	Protein phosphatase 1/2A
ROS	Reactive oxygen species
RyR2	Cardiac ryanodine receptor 2
SERCA2a	Sarco/endoplasmic reticulum Ca^2+^-ATPase 2a
SOICR	Store-overload-induced Ca^2+^ release
SR/ER	Sarcoplasmic/endoplasmic reticulum
T-tubule	Transverse tubule
